# Inhibition of gamma-secretase activity without interfering in Notch signalling decreases inflammatory response in patients with cutaneous leishmaniasis

**DOI:** 10.1080/22221751.2021.1932608

**Published:** 2021-06-16

**Authors:** Maurício T. Nascimento, Mônica Franca, Augusto M. Carvalho, Camila F. Amorim, Fábio Peixoto, Daniel Beiting, Phillip Scott, Edgar M. Carvalho, Lucas P. Carvalho

**Affiliations:** a Laboratório de Pesquisas Clínicas; Instituto Gonçalo Moniz, FIOCRUZ, Salvador, Brazil; b Serviço de Imunologia, Complexo Hospitalar Prof. Edgard Santos, Universidade Federal da Bahia, Salvador, Brazil; c Instituto de Ciências e Saúde Universidade Federal da Bahia, Salvador, Brazil; d Department of Pathobiology, University of Pennsylvania, Philadelphia, PA, USA; e Instituto Nacional de Ciências e Tecnologia-Doenças Tropicais, Salvador, Brazil

**Keywords:** Cutaneous leishmaniasis, Notch signalling, cytokines, inflammatory response, Leishmania braziliensis

## Abstract

Cutaneous leishmaniasis (CL) patients present an exacerbated inflammatory response associated with tissue damage and ulcer development. Increasing numbers of patients have exhibited treatment failure, which remains not well understood. We hypothesized that adjuvant anti-inflammatory therapy would benefit CL patients. The aim of the present study was to investigate the contribution of Notch signalling and gamma-secretase activity to the inflammatory response observed in CL patients. Notch signalling is a molecular signalling pathway conserved among animal species. Gamma-secretase forms a complex of proteins that, among other pathways, modulates Notch signalling and immune response. We found that Notch 1 cell receptor signalling protects against the pathologic inflammatory response, and JLK6, a gamma-secretase inhibitor that does not interfere with Notch signalling, was shown to decrease the *in-vitro* inflammatory response in CL. Our data suggest that JLK6 may serve as an adjuvant treatment for CL patients.

## Introduction

*Leishmania braziliensis* infection may lead to the development of cutaneous leishmaniasis (CL), the most prevalent clinical form of tegumentary leishmaniasis. CL lesions are characterized by an intense inflammatory infiltrate, high levels of pro-inflammatory cytokines TNF and IL-1β, and few parasites [1–6]. The exacerbated inflammatory response observed in these individuals is associated with ulcer development and disease progression [2,3,5,7]. Therapeutic failure in CL remains high, spanning from 30% to 70%, depending on the medication used for treatment and phase of the disease [8–11]. Thus, the search for novel adjuvant therapies, e.g. drugs that decrease inflammation without interfering in parasitic burden, is crucial.

The contribution of cytokines TNF and IL-1β in tissue damage arising from tegumentary leishmaniasis has been well-described [4,5,12,13]. The use of Pentoxifylline, a drug that decreases TNF production, as adjuvant therapy for mucosal leishmaniasis patients, the most severe form of tegumentary leishmaniasis, was observed to reduce time to healing and treatment relapse [14]. Recently, studies have documented the association of high levels of IL-1β with CL severity, and reported decreased levels upon cure [5,12,15,16]. Importantly, in humans, neither TNF nor IL-1β have been shown to participate in parasite killing, making the down-regulation of these cytokines an attractive approach for adjuvant therapy in tegumantary leishmaniasis, especially considering high reported rates of therapeutic failure [5].

The well-conserved signalling pathway mediated by Notch receptors controls a range of cell functions in mammalians, including cell fate, cytokine production, cell activation and proliferation [17–20]. Notch signalling takes place through the binding of ligands, Delta-like or Jagged, to Notch receptors, which are then cleaved by the gamma-secretase protease complex. The Notch intracellular domain (NICD) translocates to the nucleus, binds to the mastermind transcriptional activator and other co-activators, and induces the transcription of Notch target genes [21]. Interestingly, some gamma-secretase inhibitors, such as JLK6, were not found to influence Notch signalling, unveiling a non-canonical Notch signalling pathway not dependent on gamma-secretase activity [22]. The contribution of Notch signalling to mononuclear and T cell activation, as well as cytokine secretion, is well known [17–20,23–26]. Using an *L. major*-resistant mouse model of CL, it was documented that Notch 1 and 2 signalling is essential for IFN-*γ* secretion by CD4+ T cells in addition to parasite killing; however, the influence of this signalling on the secretion of proinflammatory cytokines was not investigated [27]. The expression of Notch receptors and Delta-like ligands was increased in CL lesions, particularly in patients that responded poorly to treatment, suggesting a role for Notch signalling in the promotion of an inflammatory response against CL [28]. To date, no functional analysis has been performed in human CL in an attempt to address the contribution of Notch signalling or gamma-secretase activity with respect to proinflammatory cytokine production, disease expression and parasite killing. To investigate this, we recruited CL patients and performed *in vitro* functional studies. Our results show that the expression of presinilin, a protein present in the gamma-secretase complex, was increased in CL lesions, and that the *in vitro* blockade of gamma-secretase activity decreased the inflammatory response without interfering in the parasite burden of mononuclear phagocytes.

## Materials and methods

### Subjects

This cross-sectional study was approved by the Institutional Review Board of the Professor Edgard Santos University Hospital Complex (HUPES-UFBA) (protocol no. 25/12) and the Brazilian Commission of Ethics in Research (612.907). All subjects provided written informed consent. This study was conducted in accordance with the Declaration of Helsinki and subsequent revisions. A total of 36 CL patients were recruited from an area endemic for leishmaniasis – Corte de Pedra, Bahia-Brazil. All patients included had no mucosal disease or diabetes mellitus. Pregnant women, patients older than 60 and younger than 18 years old were also excluded. Diagnostic criteria consisted of the presence of an ulcerated skin lesion, with no evidence of mucosal involvement, and the detection of *L. braziliensis* DNA by PCR. The control group consisted of 5 healthy subjects (HS), living in a non-endemic area of the same state, without any reported exposure to *Leishmania.* All CL patients underwent clinical evaluations prior to beginning treatment.

### RNA Sequencing

Unbiased RNA sequencing was performed on CL lesion and healthy subjects skin by our group, as previously described [29]. Raw sequence data is available on the Gene Expression Omnibus (GEO, accession # GSE127831).

### Parasite cultures

An isolate of *L. braziliensis* (MHOM/BR/LTCP11245) was obtained from a skin lesion of a CL patient and identified as *L. braziliensis* by multilocus enzyme electrophoresis [30]. Following isolation, parasites were cryopreserved in frozen nitrogen until use. The parasites selected for this study were not previously passaged in liquid culture medium. After selection, parasites were expanded in Schneider's medium (Sigma-Aldrich, St Louis, MO) supplemented with 20% heat-inactivated fetal bovine serum (FBS), 1% L-Glutamine, penicillin (100 U/mL) and streptomycin (100 µg/mL) (Thermo fisher scientific, NY, USA).

### Soluble *Leishmania* Antigen (SLA)

SLA was prepared from an isolate of *L. braziliensis* as previously described [31]. Briefly, promastigotes were re-suspended in lysing solution (Tris, HCL, EDTA and leupeptin), immersed in liquid nitrogen, and subsequently thawed at 37°C. After the freeze–thaw procedure, parasites were sonicated and then centrifuged at 14,000 × g. The supernatant was filtered and assayed for protein concentrations, tested for endotoxins using the Limulus amebocyte lysate test (Thermo fisher scientific, NY, USA), and used at a concentration of 5 μg/ml.

### Peripheral blood mononuclear cell cultures and biopsies

Peripheral blood mononuclear cells (PBMC) were isolated from heparinized venous blood by Ficoll-Paque (GE Healthcare, Chicago, IL) gradient centrifugation (1450 RPM). After washing in saline, cell concentrations were adjusted to 3 × 10^6^ cells in 1 ml of RPMI-1640 (Thermo fisher scientific, NY, USA) supplemented with 10% FBS (Thermo fisher scientific, NY, USA), penicillin (100 U/mL) and streptomycin (100 µg/mL) (Thermo fisher scientific, NY, USA). PBMCs were dispensed into 24-well plates and incubated at 37°C under 5% CO_2_ for 72 h in the presence or absence of SLA (5 µg/ml), anti-Notch-1 (20 μg/ml) (Thermo fisher scientific, NY, USA), anti-Notch-3 (20 μg/ml), DAPT (20 μM) and JLK6 (20 μM) (R&D Systems, Minneapolis, MS, USA). To determine cellular toxicity of gamma-secretase inhibitors (DAPT or JLK6), we performed a cell viability assay, MTT, and the concentration of 20 μM showed not to be toxic to PBMC.

Biopsies from *L. braziliensis*-infected patients and HS were performed using a 4-mm punch and cultured in complete RPMI media at 37°C, 5% CO_2_ for 72 h in the presence or absence of JLK6 (20 μM) (R&D Systems, Minneapolis, MS, USA). Supernatants were collected from PBMC and biopsy cultures and stored at −70°C until the time of cytokine quantification by ELISA (R&D Systems, Minneapolis, MS, USA), in accordance with manufacturer instructions. Non-stimulated cells did not produce relevant amounts of cytokines. Results are expressed in pg/ml.

### Monocyte cultures

Monocytes were purified from PBMCs by negative selection using MACS columns (Miltenyi Biotec, CA, USA). Monocyte were prepared following a method previously described by our laboratory to yield a purity of 99%, then characterized by flow cytometry as CD14^+^CD3^−^CD19^−^ [32]. Briefly, PBMCs were separated using a Ficoll-Paque (GE Healthcare, Chicago, IL) gradient, placed in 24-well plates at the concentration of 5 million PBMCs/well in 1 mL of RPMI 1640 supplemented with 10% human AB serum plus penicillin (100 U/mL) and streptomycin (100 µg/mL), and after 5 h of incubation at 37°C, 5% CO_2_, monocytes were separated by adherence to plastic. Next, cells infected or not with *L. braziliensis* (MOI 5:1), and stimulated or not with 20 μM of JLK6, RPMI-1640 (Thermo fisher scientific, NY, USA) supplemented with 10% FBS (Thermo fisher scientific, NY, USA), penicillin (100 U/mL) and streptomycin (100 µg/mL) (Thermo fisher scientific, NY, USA) at incubation times of 37°C, 5% CO_2_ for 2, 48 or 72 h. At each time point, infection rate and parasite burden were evaluated by optical microscopy.

## Results

**Figure 1.**
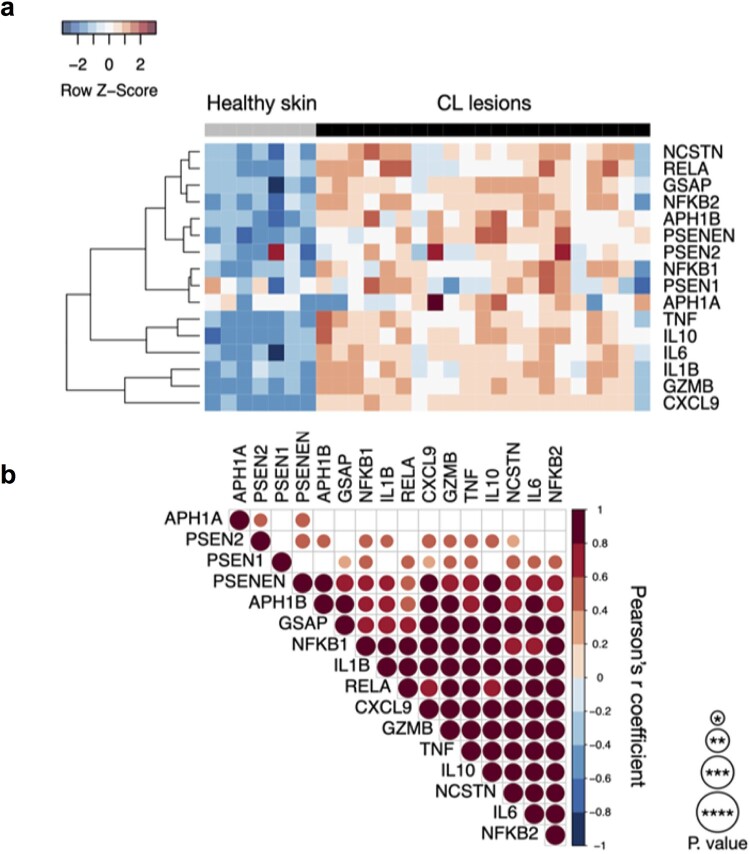
CL patients exhibit high abundance of components of gamma-secretase complex and inflammatory response genes expression in active lesions. (A) Unbiased RNASeq was performed on skin from 7 Healthy Subjects and lesion from 21 CL patients. Heatmap columns and rows represent each individual and gene, respectively. Heatmap colour reflects z-scores of gene abundance across samples. (B) Gene expression from gamma-secretase complex correlates with the inflammatory response in active lesions. Data from RNASeq (21 CL lesions) was used for correlation matrix between components of gamma-secretase complex and *NFKB1, NFKB2, RELA, TNF, IL6, IL1B, IL10, CXCL9,* and *GZMB* genes. Pearson's test was used for correlation statistical analysis and *p* value is represented according to the size of the circles.; *P < 0.05, **P < 0.01, ***P < 0.001 and ****P < 0.0001.

Gamma-secretase activity induces the cleavage of Notch receptors, which activates the Notch signalling pathway. To investigate the participation of Notch signalling components in CL we assessed unbiased gene expression in lesions from CL patients and HS. Our results show that the expression of genes involved in gamma-secretase activity, as *APH1A, APH1B, GSAP, NCSTN, PSENEN, PSEN1,* and *PSEN2,* as well as those involved in inflammation (*NFKB1, NFKB2, RELA, TNF, IL6, IL1B, IL10, CXCL9, and GZMB)* and regulation of immune response, IL-10, were increased in CL lesion when compared to healthy skin (Figure 1(a)). The expression of most of genes involved in gamma-secretase activity positively correlated with genes involved in inflammation (Figure 1(b)).

**Figure 2.**
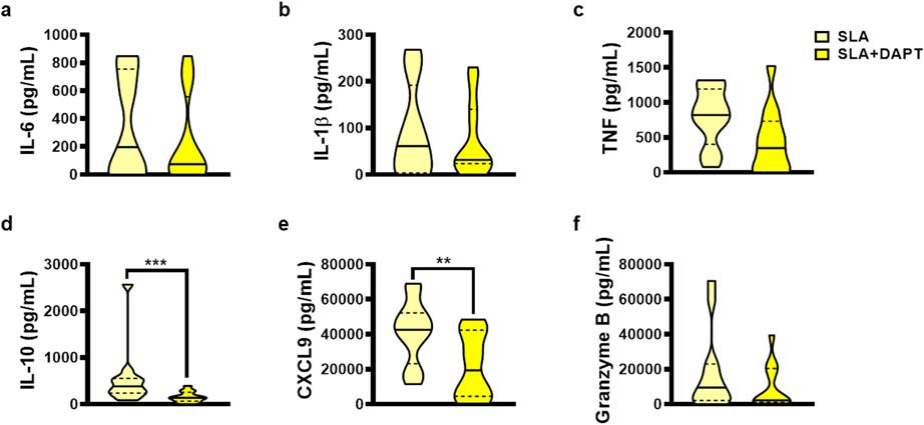
Non-selective gamma-secretase inhibitor (DAPT) decreases inflammatory-associated proteins production from CL patients in response to *Leishmania* antigens. PBMC from CL patients (*n* = 12) were cultured in presence or absence of SLA (5 ug/mL) and DAPT (20 µM) for 72 h. The levels of IL-6, IL-1β, TNF, IL-10, CXCL9, and granzyme B were determined in culture supernatants, by ELISA. The black line on the violin plot represents the percentile 50th and the dashed lines, 25th and 75th percentiles, respectively. Statistical analyses were performed using the Wilcoxon test **P < 0.01 and ***P < 0.001.

To investigate the role of gamma-secretase activity in Notch signalling, we stimulated PBMCs cultures from CL patients with SLA in the presence of DAPT, a gamma-secretase and Notch signalling inhibitor, and then assessed levels of molecules associated with inflammation in CL (IL-6, IL-1β, TNF, CXCL9, granzyme B, and IFN-γ), and the regulatory cytokine IL-10. Inhibition of gamma-secretase activity by the non-selective inhibitor DAPT was observed to decrease the production of CXCL9 and IL-10 in PBMC cultures from CL patients (Figure 2).

**Figure 3.**
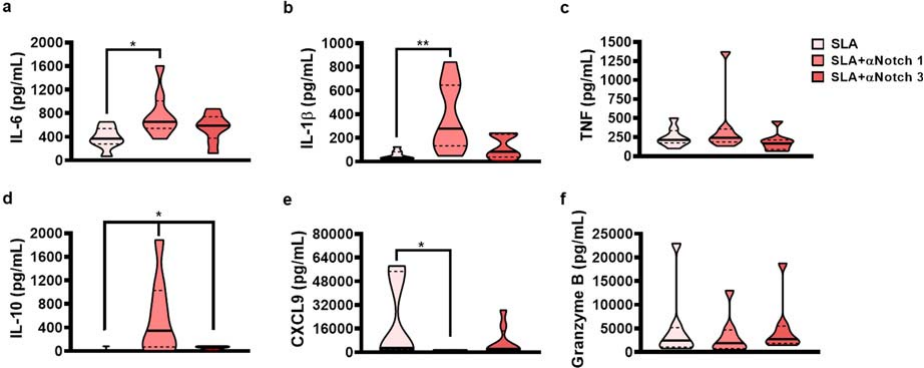
The neutralization of Notch 1 receptor increase production of proinflammatory cytokines from CL patients. PBMC from CL patients (*n* = 8) were cultured in presence or absence of SLA (5 ug/mL), anti-Notch 1 (20 µg/mL) and anti-Notch 3 (20 µg/mL) for 72 h. The levels of IL-6, IL-1β, TNF, IL-10, CXCL9, and granzyme B were determined in culture supernatants, by ELISA. The black line on the violin plot represents the percentile 50th and the dashed lines, 25th and 75th percentiles, respectively. Statistical analyses were performed using the Wilcoxon test *P < 0.05 and **P < 0.01.

Signalling through Notch-1 in macrophages increases the production of proinflammatoy cytokines by these cells and Notch-3 is known to be up-regulated in inflammatory macrophages [23,33]. To examine the role of Notch-1 and -3 in the immune response against CL, we stimulated PBMCs from CL patients with SLA in the presence of Notch-1 and -3 neutralizing antibodies. While the neutralization of Notch-1 and -3 was not shown to effect TNF and granzyme B production, surprisingly, the blockade of Notch-1 increased SLA-induced IL-6, IL-1β and IL-10, and decreased CXCL9 production (Figure 3). These findings suggest that Notch-1 signalling inhibits the production of IL-1β and IL-6 in PBMCs, which would confer a benefit to CL patients. These results were unexpected, since available literature data show that signalling though Notch 1 receptor increases proinflammatory response by macrophages [23].

**Figure 4.**
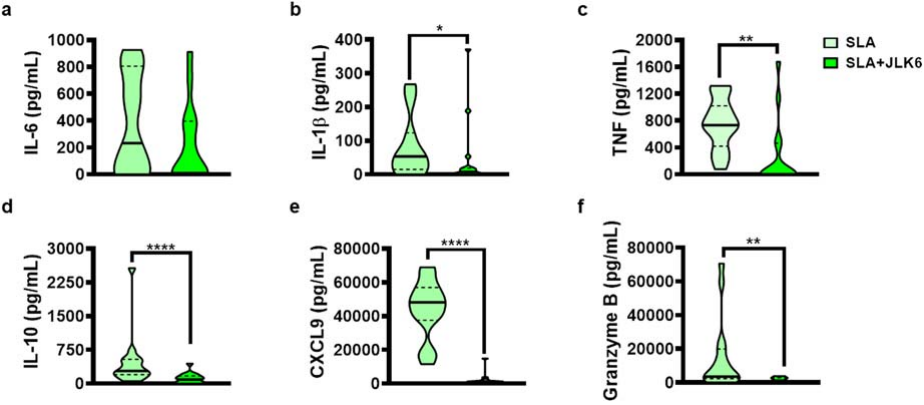
Selective gamma-secretase inhibitor (JLK6) decreases pro-inflammatory cytokine production from CL patients. PBMC from CL patients (*n* = 15) were cultured in presence or absence of SLA (5 ug/mL) and JLK6 (20 µM) for 72 h. The levels of IL-6, IL-1β, TNF, IL-10, CXCL9, and granzyme B were determined in culture supernatants, by ELISA. The black line on the violin plot represents the percentile 50th and the dashed lines, 25th and 75th percentiles, respectively. Statistical analyses were performed using the Wilcoxon test *P < 0.05, **P < 0.01 and ****P < 0.0001.

**Figure 5.**
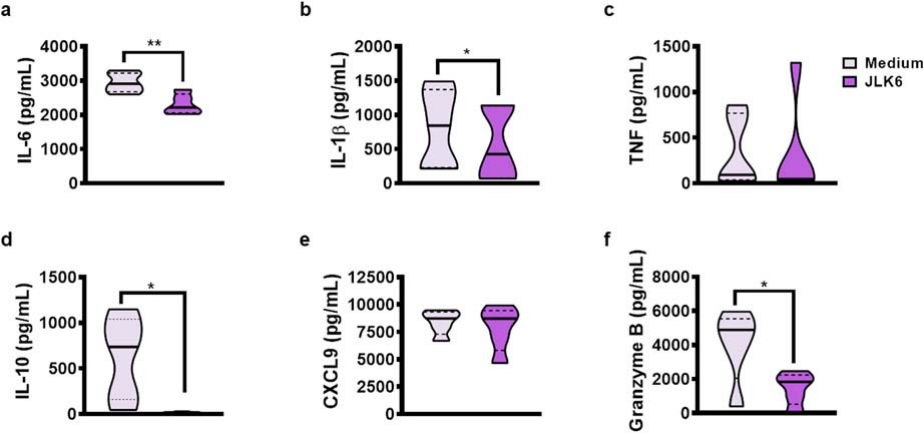
JLK6 downregulates pro-inflammatory cytokines production by lesion cells from CL patients. *L. braziliensis* lesions skin biopsies from CL patients (*n* = 5) were cultured in presence or absence of JLK6 (20 uM) for 72 h The levels of IL-6, IL-1β, TNF, IL-10, CXCL9 and granzyme B were determined in culture supernatants, by ELISA. The black line on the violin plot represents the percentile 50th and the dashed lines, 25th and 75th percentiles, respectively. Statistical analyses were performed using the Wilcoxon test *P < 0.05 and **P < 0.01.

Notch signalling can also occur in the absence of gamma-secretase activity. As the inhibition of gamma-secretase produced an opposite effect than Notch-1 and -3 on the production of proinflammatory cytokines, we chose to employ a gamma-secretase inhibitor (JLK6) that does not interfere with Notch signalling [22]. Accordingly, PBMCs stimulated with JLK6 produced lower levels of SLA-induced TNF, IL-1β, IL-10, CXCL9 and granzyme B (Figure 4). To investigate the anti-inflammatory effects of JLK6 in lesion environment, we cultured cells from CL patients’ lesions in presence of JLK6. Our results show that inhibition of gamma-secretase activity with JLK6 decreased levels of IL-6, IL-1β, granzyme B and IL-10 in cultures of lesion cells (Figure 5).

**Figure 6.**
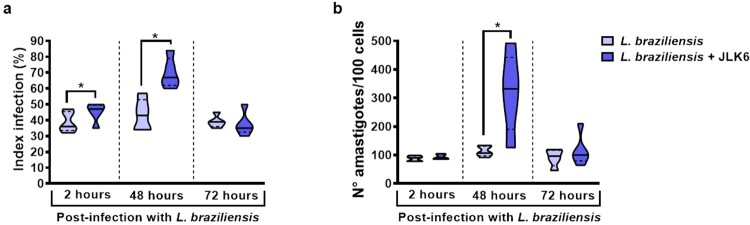
JLK6 does not affect *L. braziliensis* killing by monocytes from healthy subjects after 72 h. Monocytes from HS (*n* = 5) were infected with *L. braziliensis* in stationary phase (ratio 5:1) and cultured in presence or absence of JLK6 (20 µM) for 2, 48 and 72 h. (A) Frequency of infected cells. (B) Number of *Leishmania* amastigotes/100 monocytes. The black line on the violin plot represents the percentile 50th and the dashed lines represent the 25th and 75th percentiles, respectively. Statistical analyses were performed using the Paired t test *P < 0.05.

Considering that the observed decreases in proinflammatory cytokines may favour pathogen growth, monocyte were infected with *L. braziliensis* and stimulated or not with JLK6 to investigate the ability of these cells to kill *L. braziliensis in vitro*. The addition of JLK6 increased parasite numbers at 48 h after infection, but did not affect the number of *Leishmania* killed by human macrophages after 48 h (Figure 6).

## Discussion

Therapeutic failure rates are high in *L. braziliensis* transmission areas, reaching up to 70% depending on the clinical form of disease [9,10]. Since *L. braziliensis*-infected patients develop an exacerbated inflammatory response that leads to tissue damage, adjuvant therapy designed to decrease inflammation is desirable. For instance, patients with mucosal leishmaniasis, the most inflammatory form of tegumentary leishmanisis, benefit from the adjuvant treatment with pentoxifylline, a drug that down-regulates TNF production [14]. Interestingly, the use of pentoxifylline in combination with pentavalent antimony did not decrease time to cure or relapses in CL, suggesting that inflammatory mediators other than TNF may also play important in disease outcome [34]. Signalling through Notch receptors controls a variety of immunological process, as cell proliferation, CD4 T cell fate, macrophage activation and cytokines production [17,18,20,21,25–27]. Thus, interfering in Notch signalling as therapeutic approach may not be trivial since broad effects may be expected. Our current work investigated the role of Notch signalling on the production of inflammatory mediators in PBMCs and lesion cells from CL patients and found that JLK6, a gamma-secretase inhibitor, efficiently decreased the observed inflammatory response.

Signalling through Notch 1 in macrophages increases the production of proinflammatoy cytokines by these cells [23]. A main finding of our work is that while the blockade of Notch 1 increased proinflammatory cytokine production, the inhibition of gamma-secretase activity by JLK6, a serine protease known to inhibit γ-secretase activity of other substrates but not of Notch, led to decreased levels of inflammatory mediators in cells cultures, supporting the notion that Notch receptor signalling is indeed beneficial to CL patients. It is already known that the Gamma-secretase complex interacts with a variety of substrates, including Notch signalling, and that the JLK6 compound does not directly inhibit the presenilin-dependent gamma-secretase complex; however, the underlying mechanism behind the action of JLK6 is not completely understood. Gamma-secretase inhibitors have been tested in clinical trials. While in T-cell acute lymphoblastic leukemia/lymphoma the use of Gamma-secretase inhibitor was well tolerated, in Alzheimer patients used to decrease the production of Amyloid Beta precursor protein, which is involved in the pathogenesis of this disease, problems with tolerability and side effects have been reported [35,36]. Since most clinical trials have shown that the systemic administration of gamma-secretase inhibitors is associated with severe side effects, mainly in the intestinal tract, the use of a topical formulation of JLK6 treatment would be a safer option in the case of CL.

Among the desired effects of JLK6 on the immune response of CL patients, a decrease in the production of granzyme B was observed. Cytotoxicity has been shown to contribute to the pathogenesis of CL, and granzyme B, produced mainly by NK cells and CD8+ T lymphocytes, was previously shown to induce the production of proinflammatory cytokines in CL patients [12,37]. Moreover, we observed discrepancies in the ability of JLK6 to modulate immune response in PBMCs versus cells from lesion (e.g. CXCL9 and TNF). These differences may be due to different cell number, composition and state of activation of these cells between both sites. Also, since these individuals have open skin lesions, it cannot be ruled out the interference of other pathogens (i.e. bacteria) in the immune response at lesion site. Finally, the presence of the regulatory cytokine, IL-10, allows parasite multiplication within macrophages [38]. Here the use of JLK6 in *Leishmania*-infected monocyte-derived macrophages decreased IL-10 levels, what may improve parasite killing. However, at time point of 48 h after infection we observed increase in parasite numbers within macrophages. Future studies will be performed to investigate the effects of JLK6 in reactive oxygen species (ROS) overtime, as it has been shown that ROS production is an important mechanism to kill *Leishmania* in human macrophages [39]. Altogether, these data show that decreasing inflammatory response with JLK6 does not affect the ability of macrophage to kill *Leishmania in-vitro*.

In conclusion our current work document the advantage of blocking gamma-secretase activity without interfering in Notch signalling, making JLK6 a good candidate for adjuvant CL immunotherapy.

## Data Availability

These data is available at https://doi.org/10.6084/m9.figshare.13382723.v1

## References

[1] Bittencourt AL, Barral A. Evaluation of the histopathological classifications of American cutaneous and mucocutaneous leishmaniasis. Mem Inst Oswaldo Cruz. 1991 Jan–Mar;86(1):51–56. 10.1590/s0074-02761991000100009 1842401

[2] Carvalho LP, Passos S, Bacellar O, et al. Differential immune regulation of activated T cells between cutaneous and mucosal leishmaniasis as a model for pathogenesis. Parasite Immunol. 2007 May;29(5):251–258. 17430548 10.1111/j.1365-3024.2007.00940.x PMC2593461

[3] Carvalho LP, Passos S, Schriefer A, et al. Protective and pathologic immune responses in human tegumentary leishmaniasis. Front Immunol. 2012;3:301. 23060880 10.3389/fimmu.2012.00301 PMC3463898

[4] Passos S, Carvalho LP, Costa RS, et al. Intermediate monocytes contribute to pathologic immune response in Leishmania braziliensis infections. J Infect Dis. 2015 Jan 15;211(2):274–282. 25139016 10.1093/infdis/jiu439 PMC4334833

[5] Santos D, Campos TM, Saldanha M, et al. IL-1beta production by intermediate monocytes is associated with immunopathology in cutaneous leishmaniasis. J Invest Dermatol. 2018 May;138(5):1107–1115. 29246797 10.1016/j.jid.2017.11.029 PMC5912958

[6] Saldanha MG, Queiroz A, Machado PRL, et al. Characterization of the histopathologic features in patients in the early and late phases of cutaneous leishmaniasis. Am J Trop Med Hyg. 2017 Mar;96(3):645–652. 28115669 10.4269/ajtmh.16-0539 PMC5361539

[7] Campos TM, Costa R, Passos S, et al. Cytotoxic activity in cutaneous leishmaniasis. Mem Inst Oswaldo Cruz. 2017 Nov;112(11):733–740. 29091132 10.1590/0074-02760170109 PMC5661895

[8] Costa RS, Carvalho LP, Campos TM, et al. Early cutaneous leishmaniasis patients infected with Leishmania braziliensis express increased inflammatory responses after antimony therapy. J Infect Dis. 2018 Feb 14;217(5):840–850. 29216363 10.1093/infdis/jix627 PMC5853895

[9] Machado P, Araujo C, Da Silva AT, et al. Failure of early treatment of cutaneous leishmaniasis in preventing the development of an ulcer. Clin Infect Dis. 2002 Jun 15;34(12):E69–E73. 12032913 10.1086/340526

[10] Unger A, O'Neal S, Machado PR, et al. Association of treatment of American cutaneous leishmaniasis prior to ulcer development with high rate of failure in northeastern Brazil. Am J Trop Med Hyg. 2009 Apr;80(4):574–579. 19346378 PMC3557504

[11] Wolf Nassif P, Mello TFPDE, Navasconi TR, et al. Safety and efficacy of current alternatives in the topical treatment of cutaneous leishmaniasis: a systematic review. Parasitology. 2017 Jul;144(8):995–1004. 28367792 10.1017/S0031182017000385

[12] Novais FO, Carvalho AM, Clark ML, et al. CD8+ t cell cytotoxicity mediates pathology in the skin by inflammasome activation and IL-1β production. PLoS Pathog. 2017 Feb;13(2):e1006196. 28192528 10.1371/journal.ppat.1006196 PMC5325592

[13] Novais FO, Carvalho LP, Passos S, et al. Genomic profiling of human Leishmania braziliensis lesions identifies transcriptional modules associated with cutaneous immunopathology. J Invest Dermatol. 2015 Jan;135(1):94–101. 25036052 10.1038/jid.2014.305 PMC4268311

[14] Lessa HA, Machado P, Lima F, et al. Successful treatment of refractory mucosal leishmaniasis with pentoxifylline plus antimony. Am J Trop Med Hyg. 2001 Aug;65(2):87–89. 11508396 10.4269/ajtmh.2001.65.87

[15] Carvalho AM, Novais FO, Paixao CS, et al. Glyburide, a NLRP3 inhibitor, decreases inflammatory response and is a candidate to reduce pathology in Leishmania braziliensis infection. J Invest Dermatol. 2020 Jan;140(1):246–249e2. 31252034 10.1016/j.jid.2019.05.025 PMC7851844

[16] Charmoy M, Hurrell BP, Romano A, et al. The Nlrp3 inflammasome, IL-1β, and neutrophil recruitment are required for susceptibility to a nonhealing strain of leishmania major in C57BL/6 mice. Eur J Immunol. 2016 Apr;46(4):897–911. 26689285 10.1002/eji.201546015 PMC4828310

[17] Amsen D, Blander JM, Lee GR, et al. Instruction of distinct CD4 T helper cell fates by different notch ligands on antigen-presenting cells. Cell. 2004 May 14;117(4):515–526. 15137944 10.1016/s0092-8674(04)00451-9

[18] Robey E, Chang D, Itano A, et al. An activated form of Notch influences the choice between CD4 and CD8 T cell lineages. Cell. 1996 Nov 1;87(3):483–492. 8898201 10.1016/s0092-8674(00)81368-9

[19] Tanigaki K, Tsuji M, Yamamoto N, et al. Regulation of alphabeta/gammadelta T cell lineage commitment and peripheral T cell responses by Notch/RBP-J signaling. Immunity. 2004 May;20(5):611–622. 15142529 10.1016/s1074-7613(04)00109-8

[20] Washburn T, Schweighoffer E, Gridley T, et al. Notch activity influences the alphabeta versus gammadelta T cell lineage decision. Cell. 1997 Mar 21;88(6):833–843. 9118226 10.1016/s0092-8674(00)81929-7

[21] Andersson ER, Sandberg R, Lendahl U. Notch signaling: simplicity in design, versatility in function. Development. 2011 Sep;138(17):3593–3612. 21828089 10.1242/dev.063610

[22] Petit A, Bihel F, Alves da Costa C, et al. New protease inhibitors prevent gamma-secretase-mediated production of Abeta40/42 without affecting Notch cleavage. Nat Cell Biol. 2001 May;3(5):507–511. 11331880 10.1038/35074581

[23] Monsalve E, Ruiz-Garcia A, Baladron V, et al. Notch1 upregulates LPS-induced macrophage activation by increasing NF-kappaB activity. Eur J Immunol. 2009 Sep;39(9):2556–2570. 19662631 10.1002/eji.200838722

[24] Palaga T, Buranaruk C, Rengpipat S, et al. Notch signaling is activated by TLR stimulation and regulates macrophage functions. Eur J Immunol. 2008 Jan;38(1):174–183. 18085664 10.1002/eji.200636999

[25] Song LL, Peng Y, Yun J, et al. Notch-1 associates with IKKalpha and regulates IKK activity in cervical cancer cells. Oncogene. 2008 Oct 2;27(44):5833–5844. 18560356 10.1038/onc.2008.190

[26] Weijzen S, Velders MP, Elmishad AG, et al. The Notch ligand jagged-1 is able to induce maturation of monocyte-derived human dendritic cells. J Immunol. 2002 Oct 15;169(8):4273–4278. 12370358 10.4049/jimmunol.169.8.4273

[27] Auderset F, Coutaz M, Tacchini-Cottier F. The role of Notch in the differentiation of CD4(+) T helper cells. Curr Top Microbiol Immunol. 2012;360:115–134. 22653552 10.1007/82_2012_227

[28] Rodrigues KM, Oliveira MP, Maretti-Mira AC, et al. Influence of the Notch system in the therapeutic response of American tegumentary leishmaniasis. Br J Dermatol. 2011 Jun;164(6):1228–1234. 21299543 10.1111/j.1365-2133.2011.10240.x

[29] Amorim CF, Novais FO, Nguyen BT, et al. Variable gene expression and parasite load predict treatment outcome in cutaneous leishmaniasis. Sci Transl Med. 2019 Nov 20;11(519):1–9. 10.1126/scitranslmed.aax4204 PMC7068779 31748229

[30] Cupolillo E, Grimaldi G, Jr., Momen H. A general classification of new world leishmania using numerical zymotaxonomy. Am J Trop Med Hyg. 1994 Mar;50(3):296–311. 8147488 10.4269/ajtmh.1994.50.296

[31] Teixeira R, Reed SG, Badaro R, et al. Selection of a skin test antigen for American visceral leishmaniasis. Am J Trop Med Hyg. 1986 Jan;35(1):79–85. 3946739 10.4269/ajtmh.1986.35.79

[32] Giudice A, Vendrame C, Bezerra C, et al. Macrophages participate in host protection and the disease pathology associated with Leishmania braziliensis infection. BMC Infect Dis. 2012 Mar 29;12:75. 22458474 10.1186/1471-2334-12-75 PMC3373377

[33] López-López S, Monsalve EM, Romero de Ávila MJ, et al. NOTCH3 signaling is essential for NF-κB activation in TLR-activated macrophages. Sci Rep. 2020 Sep 9;10(1):14839. 32908186 10.1038/s41598-020-71810-4 PMC7481794

[34] Brito G, Dourado M, Guimarães LH, et al. Oral pentoxifylline associated with pentavalent antimony: a randomized trial for cutaneous leishmaniasis. Am J Trop Med Hyg. 2017 May;96(5):1155–1159. 28500815 10.4269/ajtmh.16-0435 PMC5417210

[35] Mikulca JA, Nguyen V, Gajdosik DA, et al. Potential novel targets for Alzheimer pharmacotherapy: II. update on secretase inhibitors and related approaches. J Clin Pharm Ther. 2014 Feb;39(1):25–37. 24313554 10.1111/jcpt.12112

[36] Toyn JH, Ahlijanian MK. Interpreting Alzheimer’s disease clinical trials in light of the effects on amyloid-β. Alzheimers Res Ther. 2014;6(2):14. 25031632 10.1186/alzrt244 PMC4014014

[37] Campos TM, Novais FO, Saldanha M, et al. Granzyme B produced by natural killer cells enhances inflammatory response and contributes to the immunopathology of cutaneous leishmaniasis. J Infect Dis. 2020 Mar 2;221(6):973–982. 31748808 10.1093/infdis/jiz538 PMC7050991

[38] Kane MM, Mosser DM. The role of IL-10 in promoting disease progression in leishmaniasis. J Immunol. 2001 Jan 15;166(2):1141–1147. 11145695 10.4049/jimmunol.166.2.1141

[39] Novais FO, Nguyen BT, Beiting DP, et al. Human classical monocytes control the intracellular stage of Leishmania braziliensis by reactive oxygen species. J Infect Dis. 2014 Apr 15;209(8):1288–1296. 24403561 10.1093/infdis/jiu013 PMC3969552

